# Taxonomic and Functional Microbial Signatures of the Endemic Marine Sponge *Arenosclera brasiliensis*

**DOI:** 10.1371/journal.pone.0039905

**Published:** 2012-07-02

**Authors:** Amaro E. Trindade-Silva, Cintia Rua, Genivaldo G. Z. Silva, Bas E. Dutilh, Ana Paula B. Moreira, Robert A. Edwards, Eduardo Hajdu, Gisele Lobo-Hajdu, Ana Tereza Vasconcelos, Roberto G. S. Berlinck, Fabiano L. Thompson

**Affiliations:** 1 Instituto de Química de São Carlos, Universidade de São Paulo, São Carlos, Brazil; 2 Instituto de Biologia, Universidade Federal do Rio de Janeiro, Rio de Janeiro, Brazil; 3 Department of Computer Science, San Diego State University, San Diego, United States of America; 4 Centre for Molecular and Biomolecular Informatics, Nijmegen Centre for Molecular Life Sciences, Radboud University Nijmegen Medical Centre, Nijmegen, The Netherlands; 5 Division of Mathematics and Computer Science, Argonne National Laboratory, Argonne, United States of America; 6 Departamento de Invertebrados, Museu Nacional, Universidade Federal do Rio de Janeiro, Rio de Janeiro, Brazil; 7 Instituto de Biologia Roberto Alcantara Gomes, Universidade do Estado do Rio de Janeiro, Rio de Janeiro, Brazil; 8 Laboratório Nacional de Computação Científica, Petrópolis, Brazil; J. Craig Venter Institute, United States of America

## Abstract

The endemic marine sponge *Arenosclera brasiliensis* (Porifera, Demospongiae, Haplosclerida) is a known source of secondary metabolites such as arenosclerins A-C. In the present study, we established the composition of the *A. brasiliensis* microbiome and the metabolic pathways associated with this community. We used 454 shotgun pyrosequencing to generate approximately 640,000 high-quality sponge-derived sequences (∼150 Mb). Clustering analysis including sponge, seawater and twenty-three other metagenomes derived from marine animal microbiomes shows that *A. brasiliensis* contains a specific microbiome. Fourteen bacterial phyla (including *Proteobacteria*, *Cyanobacteria*, *Actinobacteria*, *Bacteroidetes*, *Firmicutes* and *Cloroflexi*) were consistently found in the *A. brasiliensis* metagenomes. The *A. brasiliensis* microbiome is enriched for *Betaproteobacteria* (e.g., *Burkholderia*) and *Gammaproteobacteria* (e.g., *Pseudomonas* and *Alteromonas*) compared with the surrounding planktonic microbial communities. Functional analysis based on **R**apid **A**nnotation using **S**ubsystem **T**echnology (RAST) indicated that the *A. brasiliensis* microbiome is enriched for sequences associated with membrane transport and one-carbon metabolism. In addition, there was an overrepresentation of sequences associated with aerobic and anaerobic metabolism as well as the synthesis and degradation of secondary metabolites. This study represents the first analysis of sponge-associated microbial communities via shotgun pyrosequencing, a strategy commonly applied in similar analyses in other marine invertebrate hosts, such as corals and algae. We demonstrate that *A. brasiliensis* has a unique microbiome that is distinct from that of the surrounding planktonic microbes and from other marine organisms, indicating a species-specific microbiome.

## Introduction

Sponges are probably the most primitive metazoans, with fossil records for this group dating from 635 to 750 million years ago [1]. As much as 40% of sponge wet weight may be comprised of microbes, including sponge-specific prokaryotic communities [2], [3], [4], [5], [6]. There are at least 15,000 sponge species on the planet, inhabiting different types of environments from the deep sea to riverine systems. Pioneering electron microscopy and cultivation-dependent approaches suggested the existence of three groups of sponge-associated microbes: mesohyl sponge-specific microbes, intracellular symbionts, and non-specific transient microbial communities that are shared between sponges and the surrounding water column [2]. Cultivation-independent taxonomic characterization using 16S rRNA library sequencing approaches provided a broader, cultivation-independent taxonomic characterization of sponge microbiomes and revealed significant microbial diversity that included sponge-exclusive microbes, such as the organisms classified as belonging to the candidate bacterial phylum Poribacteria [7].

The most recent studies applying massively parallel 16S rRNA gene tag sequencing, based on approximately 32,000 tag sequences (read length >125 nt), suggest that the sponge microbiome may be classified into three main groups (species-specific, variable, and core) [8], [9]. Species-specific microbes comprise 72% of the taxa detected in sponges, whereas only 2% of the detected taxa correspond to the core found in several species of sponges. The five Mediterranean sponges *Aplysina aerophoba*, *Aplysina cavernicola*, *Ircinia variabilis*, *Petrosia ficiformis*, and *Pseudocorticium jarrei* share a core microbiome containing operational taxonomic units (OTUs) from the phyla *Acidobacteria*, *Chloroflexi*, *Nitrospira*, *Poribacteria*, and *Proteobacteria* [8]. The phylum *Chlamydiae* appears to be found only in association with *A. cavernicola*, whereas the phylum *Lenthisphaerae* occurs only in association with *I. variabilis* [8]. The core community could represent globally distributed microbes that are horizontally acquired from the environment by a sponge, whereas the species-specific community could consist of microbes with a distribution restricted (endemic) to a single sponge species that are vertically acquired from the progenitor [9]. A recent study investigating *Cymbastela concentrica* microbial diversity helped to further elucidate possible genetic mechanisms involved in the establishment of species-specific microbiomes [10]. The authors generated 190,623 shotgun sequences (92.6 Mbp) and 3,545 16S rRNA sequences (>1,200 bp in length per sequence). *Gammaproteobacteria*, *Phyllobacteriaceae*, *Sphingomondales*, *Neisseriales*, and *Nitrospiraceae* constituted the vast majority of the microbiome of *C. concentrica*. Based on 16S rRNA identification (97% identity cutoff), only thirty-four different OTUs were common between *C. concentrica* and the surrounding seawater, supporting the idea of selection for a specific microbiome, possibly consisting of several (though non-culturable) bacteria. Functional analysis performed via shotgun sequencing using the COG database suggested that the majority of the detected genes (>85%) belong to bacteria. The authors also found a large number of sequences identified as transposable insertion elements. The sponge metagenomes contained a greater number of sequences identified as COG0610 (restriction enzymes, type I helicase) and COG1715 (restriction endonuclease) than the surrounding seawater. Both COG groups include specific DNA modification and restriction systems in bacteria, and therefore, the authors hypothesized that this would facilitate horizontal DNA exchange between sponge microorganisms and protect against DNA exchange with planktonic organisms in the surrounding seawater [10].

Despite the significant advances in the study of sponge microbial diversity in the Mediterranean and Pacific, very little is known concerning the functional and taxonomic diversity of sponge microbiomes in the South Atlantic. A recent 16S rRNA sequence-based study in Rio de Janeiro (southeastern Atlantic region) generated 133 bacterial sequences from the sponges *Hymeniacidon heliophila* and *Polymastia janeirensis* [14]. These two sponge species appear to share several bacterial taxa affiliated with *Cyanobacteria* and *Proteobacteria*. *Alphaproteobacteria* was the most abundant group. An analysis of 254 archaeal 16S rRNA partial sequences from the sponges *Hymeniacidon heliophila*, *Polymastia janeirensis*, *Paraleucilla magna*, and *Petromica citrina* suggested that *Crenarchaeota*, a phylum that is well represented in sequence databases and is related to the *Thaumarchaeotal* sponge symbiont *Cenarchaeum symbiosum*, dominated the archaeal microbiome of the sponge *P. citrina* [15].

Sponges belonging to the order Haplosclerida (class Demospongiae) are a rich source of polycyclic alkylpiperidine alkaloids [16], whose microbial secondary metabolites remain unknown. These secondary metabolites may be important in regulating the composition of the sponge microbiome. *Arenosclera brasiliensis* is a shallow-water haplosclerid sponge endemic to Rio de Janeiro State (Brazil) that colonizes rocky shores in the Búzios and Arraial do Cabo areas [17]. *Arenosclera brasiliensis* is white, cream or beige in color and exhibits a soft texture. It is approximately 15 cm wide, 7 cm long and 12 cm in height. It has circular or oval-shaped osculuns (9 mm in diameter) and spicules of up to 110 µm in length. *A. brasiliensis* contains arenosclerins A-C, which are novel cytotoxic, antimicrobial alkylpiperidine alkaloids, for which the actual biosynthetic origin (whether they are produced by microbes) is still unknown [18], [19], [20]. In the present, study we determined the composition of the *A. brasiliensis* microbiome and the major metabolic pathways in this organism. This is the first metagenomic characterization of the Brazilian endemic sponge *A. brasiliensis*. We used 454 shotgun pyrosequencing to generate more than 640,000 high-quality sponge-derived sequences (∼150 Mb), representing an unprecedented amount of metagenomic information for sponges of the southeastern Atlantic.

## Materials and Methods

### Sponge and Water Sampling

On two occasions (May 2010 and January 2011), three specimens of *A. brasiliensis* were collected at a depth of ∼5 m via SCUBA diving off the rocky coast of João Fernandinho’s Beach on the Armação dos Búzios peninsula in the state of Rio de Janeiro (22°44′49″S/41°52′54″W), Brazil. The specimens were transported to the laboratory in ∼20 L of temperature-conditioned (∼24°C) aerated seawater. The samples were processed, and DNA was extracted on the same day (see below). We collected specimens at a distance of at least 5 meters from each other. During each expedition, a volume of 8 L of water from the water column surrounding the sponges was also collected. From these samples, 4 replicates (2 L each) of seawater were pre-filtered through a 20 µm nylon filter followed by a 5 µm filter and, finally, using a 0.22 µm polyethersulfone Sterivex™-GP filter (Millipore, Billerica, MA, USA,) for sampling planktonic microorganisms (approximately 2 h of filtering at no more than 45 psi). The Sterivex filters containing microbial cells were immediately filled with 2 ml of SET buffer and stored in liquid nitrogen [21].

### DNA Extraction, Pyrosequencing and Annotation

In the laboratory, individual sponges were transferred to a container filled with 250 ml of sterile seawater and left for 5–10 minutes to wash away unassociated microorganisms by recirculating the water. The sponge tissue was dried via compression between stacks of sterile paper towels and then dissected with a scalpel into 0.5–1 cm^3^ pieces, carefully removing any macroscopic organisms associated with the sponge tissue (i.e., nematodes or polychaetes). Approximately 1 g of processed tissue was then frozen using liquid nitrogen and ground, and DNA was extracted and purified using 4 M guanidine hydrochloride, 50 mM Tris-HCl pH 8.0, 0.05 M EDTA, 0.5% sodium-N’-lauroylsarcosine, and 1% β-mercaptoethanol, followed by a phenol/chloroform step, as described previously [22]. The DNA from microorganisms retained in the Sterivex filters was extracted as previously described [21]. Approximately 0.5 µg of total DNA from *A. brasiliensis* tissue or Sterivex-filtered microbes was then sequenced at the Laboratório Nacional de Computação Científica (LNCC) (from February to April of 2011) using 454-pyrosequencing methodology [23] with GS-FLX TITANIUM chemistry (Roche Applied Science). Unassembled 454-generated sequencing reads were annotated using the **M**eta-**G**enome **R**apid **A**nnotation using **S**ubsystems **T**echnology (MG-RAST) server [24], version 3.0, utilizing (SEED) Subsystems Technology and the GenBank database for *functional* and *organismal* classifications, respectively. All BLAST queries were conducted with a cutoff *E*-value of 10^−5^ and a minimum alignment length of 50 bp.

**Figure 1:**
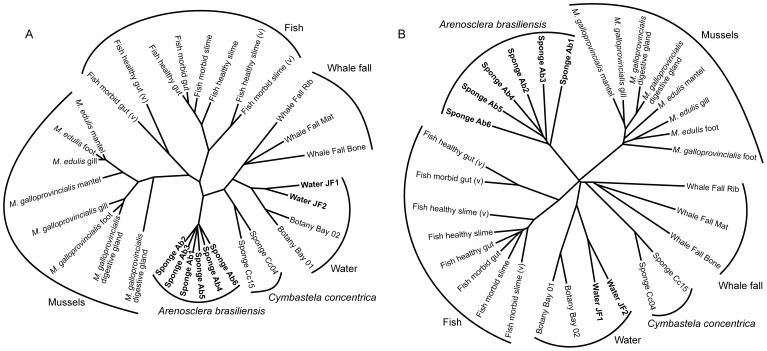
Comparison of the metagenomes of marine organism-associated microbiomes. (A) Cladogram representing the similarities in the taxonomic profiles of the best BLASTn hits for the reads. (B) Cladogram representing the fraction of cross-contigs (i.e., shared contigs that contain reads from two or more metagenomes) after cross-assembly. We used available shotgun metagenomic samples from the Australian sponge *Cymbastela concentrica* [PMID: 20520651], healthy and morbid fish [PMID: 18337718], the mussel species *Mytilus galloprovincialis* and *M. edulis* [PMID: 20111607] and a whale fall [PMID: 15845853]. Four water samples are included for comparison: two from this study and two from a study on *C. concentrica* [PMID: 20520651]. Cladograms were created with BioNJ [34], as explained in the Materials and Methods section.

To determine the functional contributions of specific bacterial groups, the *functional* annotations (hierarchical level 1) were separated individually using the MG-RAST Workbench tool and submitted for *organismal* classifications (*E*-value = 10^−5^; and minimum alignment length = 50 bp). A table compiling the functional contributions of the organisms in each sponge sample (Ab1 to Ab6) was then generated and uploaded to the **St**atistical **A**nalysis of **M**etagenomic **P**rofiles (STAMP) software package, version 2.0.0 [25]. Statistical tests were conducted treating each organismal group as a sample, as explained below (*Statistical analysis*).

Metagenomic samples were also queried via BLASTn [26] searches against the genome of the haplosclerid sponge *Amphimedon queenslandica* [27]. BLAST queries were conducted with the same stringency parameters as described above.

### Statistical Analysis

The functional contributions of each organism that differed significantly (p-value <0.05) were identified via analysis of variance (ANOVA) with the Tukey-Kramer post-hoc test (confidence interval of 0.95) and the Benjamini-Hochberg multiple test false discovery rates (FDR) correction [28], [29] using STAMP. All other statistical calculations were conducted with R-2.14.0 (www.r-project.org) using the ShotgunFunctionalizeR package [30]. Rarefaction curves were derived based on each sample’s organismal classifications at the species level. Sample diversity was also estimated for the species hierarchy using Chao’s, Simpson’s, and Shannon’s diversity indices. Direct comparisons of *A. brasiliensis* (Ab) and water (JF) samples were performed using a non-parametric Poisson model. The default Benjamini-Hochberg False Discovery Rate was used to generate corrected P-values (q-values). Functional (or taxonomic) classifications with q-values <10^−5^ were considered to represent significant differences. Effects of unequal sample sizes were removed using the total number of reads assigned to each hierarchical level as an offset of each metagenome, as implemented by the program.

### Cross-assembly (crAss)

The sequencing reads from all eight metagenomes were combined with 23 other metagenomes obtained from the following marine animal microbiomes: the Australian sponge *Cymbastela concentrica* and two water samples collected at the same site [PMID: 20520651], healthy and morbid fish [PMID: 18337718], the mussel species *Mytilus galloprovincialis* and *M. edulis* [PMID: 20111607] and a whale fall [PMID: 15845853]. All of these metagenomes were cross-assembled using gsAssembler [23], and the results were visualized using the metagenome cross-assembly tool crAss (http://sf.net/p/crAss). Briefly, crAss calculates a distance matrix between all pairs of metagenomes and corrects for sample size using the SHOT formula, which has previously been used to correct for genome size when calculating phylogenetic distances between species [31], [32], [33]. This distance matrix was converted into a cladogram using BioNJ [34] and was visualized using Drawtree [35].

### Taxonomic Profiles

The sequencing reads from all thirty-one marine animal metagenomes (listed above) were queried using BLASTn searches (version 2.2.25, E-value cutoff of ≤10^−5^) [PMID: 2231712] against the GenBank NT database (January 16^th^ 2012 version) [PMID: 22144687], and the NCBI taxonomy IDs [PMID: 18940862] of the top hits were recorded. For each taxon, we counted the number of reads that mapped to it, and in instances where multiple top hits with an equal BLASTn bitscore occurred, the read was divided equally. We calculated the number of reads mapped to parent clades by cumulatively summing the reads in daughter clades. From the taxonomic profiles created in this manner (Table S1), we calculated a distance matrix based on the Wootters distance metric [36].
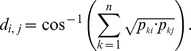



This formula is based on the fractions of reads from metagenomes *i* and *j*, represented by 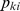 and 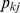, respectively, that are incorporated into contig *k*. It estimates the minimum number of jumps required to move from one distribution to another, where a jump reflects a statistical fluctuation typical of a finite sampling event. The distance is normalized so that it is independent of the sample size. The distance matrix was transformed into a cladogram using BioNJ [34].

**Figure 2:**
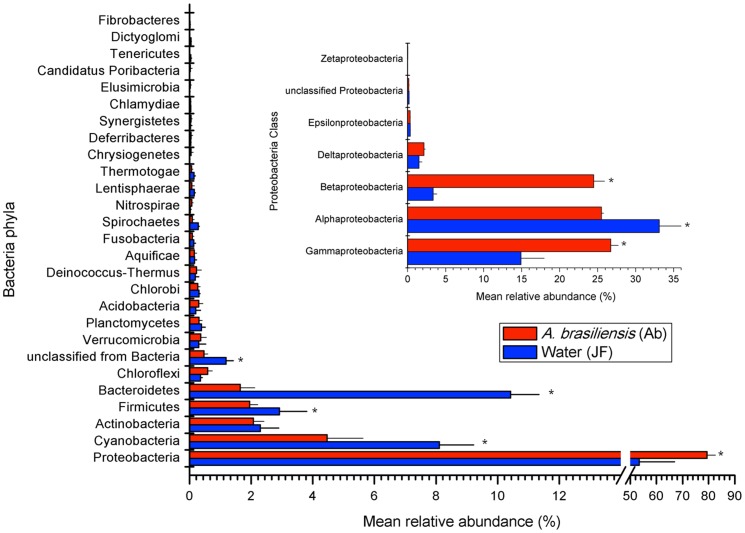
Relative abundances of bacterial phyla and proteobacterial classes (*inset*) in the sponge and seawater organismal classifications. Asterisks indicate organisms differing significantly (p-value <0.00001) between groups based on direct Poisson model-based comparisons.

**Figure 3:**
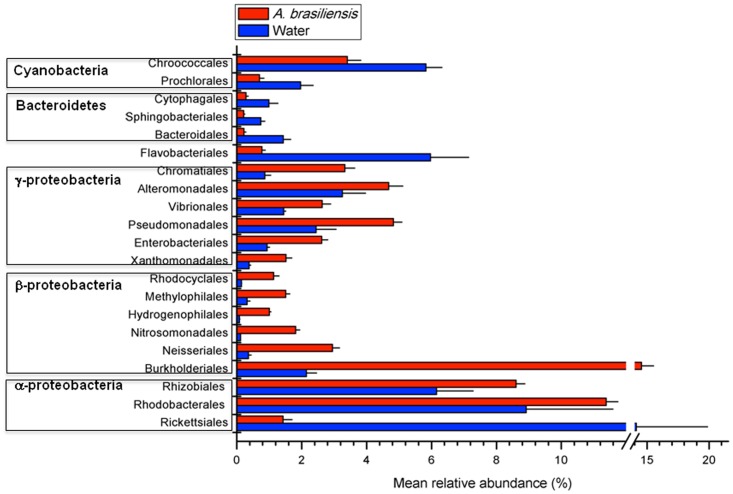
Bacterial orders that were significantly overrepresented in sponge or seawater metagenomes. Organismal classifications differing significantly (p-value <0.00001) were indicated by direct Poisson model-based comparisons of the two groups of metagenomes.

## Results

We generated approximately 674,000 (200 Mbases) high-quality shotgun metagenomic sequences (averaging 293 nt in length) from six sponge specimens (Ab1 to Ab6) and two seawater samples (JF1 and JF2) (Table S2). According to MG-RAST analyses, only 5.5% (30,427 sequences) and 8.9% (49,206 sequences) of the sponge-derived sequences were identified within the taxonomic hierarchy and subsystems, respectively (Table S2). These percentages were seven times lower than those obtained for the water-derived sequences, indicating a large reservoir of novel biodiversity in the sponge metagenomes. To detect metagenomic sequences originating from the *A. brasiliensi*s genome, the six samples from this species (Ab1 to Ab6) were compared using BLASTn to the genome of *Amphimedon queenslandica* [27], which is the only genome available for Porifera. *A. queenslandica* is a phylogenetic neighbor of *A. brasiliensis*, also belonging to the haplosclerid suborder Haplosclerina. Only ∼0.3% of *A. brasiliensis-*derived sequences matched *A. queenslandica* genomic regions (Table S2). Although this percentage is relatively low, it was ten times higher than that obtained when the water-derived data were used for comparison (∼0.03%) (Table S2).

### The Core Microbiome of *A. brasiliensis*

The majority of the sequences detected in the *A. brasiliensis* (93%) and seawater (83%) samples were identified as bacterial in origin (Figure S1A). A significant portion of this microbial diversity was covered by our sequencing efforts (Figure S1B). A complementary clustering analysis (see *Materials and Methods*) showed that the *A. brasiliensis*-derived metagenomic sequences formed a cohesive cluster that clearly separates them from the clusters formed by the microorganisms associated with other marine animals (Figure 1A and 1B). The *A. brasiliensis* clade also branched apart from the clade formed by the microbiome of the sponge *C. concentrica*, while the seawater samples collected near both sponge species (JF1, JF2 and Botany Bay 1 and 2) could be grouped into a main clade of planktonic microorganisms (Figure 1A and 1B, water clade). These results strongly indicate that *A. brasiliensis* contains a specifically associated microbial community. The abundances of sequences identified as eukaryotes (∼3–5%) and Archaea (∼1%) were similar in the sponge and seawater groups, whereas viruses were more abundant in the seawater metagenomes (Figure S1A).

The microbiome of *A. brasiliensis* was rather homogeneous across the different specimens, and a total of 26 bacterial phyla were detected (Figure 2). Fourteen phyla were found consistently in all of the metagenomes (Figure 2). *Proteobacteria*, *Cyanobacteria*, *Actinobacteria*, *Bacteroidetes*, *Firmicutes*, and *Cloroflexi* were present at abundances of ≥1%. *Planctomycetes, Verrucomicrobia, Chlorobi, Deinococcus-Thermus, Nitrospirae, Aquificae, Spirochaetes, Acidobacteria,* and *Fusobacteria* were present at abundances of ≤1% (Figure 2). *Proteobacteria* was the most abundant phylum in the sponge (∼79%) and water (∼53%) metagenomes (Figure 2). The ShotgunFunctionalizeR non-parametric Poisson model showed that sponge samples were significantly enriched for *Beta*- (∼24.5%) and *Gammaproteobacteria* (∼26.7%) sequences (adjusted p-value <0.00001), whereas *Alphaproteobacteria* sequences were overrepresented in the water metagenomes (Figure 2, *inset*). We found six *Gammaproteobacteria*, six *Betaproteobacteria* and two *Alphaproteobacteria* orders overrepresented in the sponge samples (Figure 3). *Burkholderiales* accounted for ∼15% of the sponge metagenomes (Figure 3), and *Burkholderia* was the most abundant sponge-associated bacterial genus (Table S3). Methanotrophic (*Methylophylales*) and photosynthetic (*Chromatiales*) proteobacteria were also enriched in *A. brasiliensis* metagenomes (Figure 3). Sequences identified as *Pelagibacter* (*Rickettsiales*) were more abundant in the water metagenomes (Table S3; Figures 2 and 3).

### Functional Properties of the *A. brasiliensis* Microbiome

**Figure 4:**
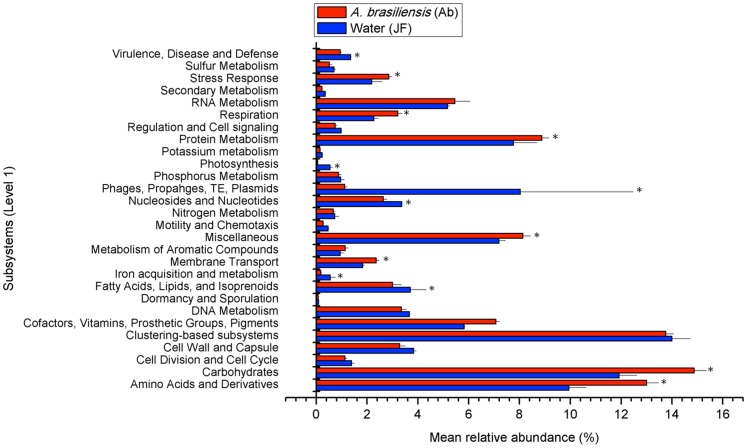
Relative abundances of subsystems (Level 1) in the sponge and seawater metagenomes. Asterisks indicate a p-value <0.00001 in direct Poisson model-based comparisons of the two groups of metagenomes.

**Figure 5:**
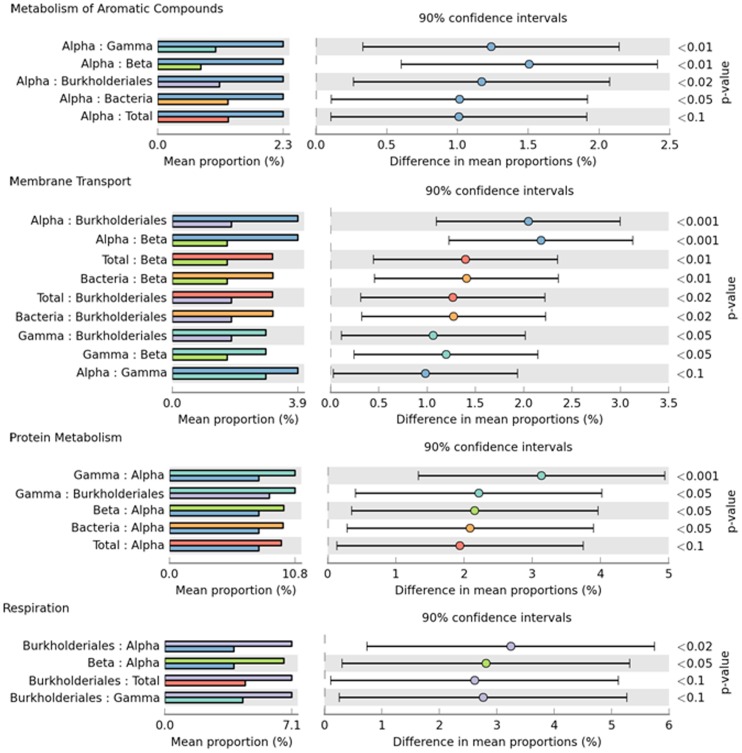
Relative functional contributions that differed significantly between the dominant proteobacterial groups. The first column shows each pair of organisms (e.g., *Alpha*- and *Gammaproteobacteria*) that differed significantly (p-value <0.05) for a subsystem (e.g., “metabolism of aromatic compounds”), detected using ANOVA for multiple groups. The percentage bars were colored as follows: *Alphaproteobacteria* (blue); *Gammaproteobacteria* (orange); *Betaproteobacteria* (green), *Burkholderiales* (purple); bacteria (light blue); and total species (red). The second column shows the detected differences with the 90% confidence interval, as calculated using the Tukey-Kramer post-hoc test.

Direct Poisson model-based comparisons between the functional classifications for the *A. brasiliensis* and seawater metagenomes showed that the relative abundances of 13 out of the 28 Level-1 subsystems (SS) were significantly different (p-value <0.00001) (Figure 4). The *A. brasiliensis* metagenomes were enriched for more than 140 functions comprising 7 Level-1 SSs (membrane transport; stress responses; respiration; cofactors, vitamins, prosthetic groups, and pigments; protein metabolism; amino acids and derivatives; and carbohydrates; Table S4 and Figure 4). The seawater metagenomes exhibited a different SS profile, showing an abundance of the virulence, disease and defense; photosynthesis; phages, prophages, transposable elements, plasmids; nucleosides and nucleotides, iron acquisition and metabolism; and fatty acids, lipids, and isoprenoid subsystems. *Alphaproteobacteria* contributed relatively more than all other groups to sequences involved in the metabolism of aromatic compounds and more than *Gamma*- and *Betaproteobacteria* to sequences encoding membrane transporters (Figure 5). In contrast, *Gammaproteobacteria* made a larger contribution to protein metabolism than *Alphaproteoabcteria* and *Burkholderiales*, whereas *Burkholderiales* made the greatest relative contribution to sequences associated with respiration (Figure 5).

## Discussion

In the present work, we aimed to analyze the diversity of microorganisms associated with the endemic shallow-water haplosclerid sponge *Arenoslcera brasiliensis*. We showed that the microbiomes of six sponge specimens collected on two occasions at an interval of eight months (May 2010 and January 2011) were highly similar. This type of close association between sponge hosts and microbes has been observed in previous investigations [4], [10]. Moreover, clustering analysis showed that the *A. brasiliensis* microbiome is distinguishable from the microbiomes present in seawater and those associated with other marine animals, including the sponge *C. concentrica*, strongly suggesting that *A. brasiliensis* exhibits a species-specific microbiome [11], [12]. The microbiome associated with *A. brasiliensis* is highly enriched for protein coding sequences assigned to the domain Bacteria (92%). It is debatable whether the low frequency of sequences assigned to Archaea (∼1% of the classifiable sequences) reflects a bias of the databases used for BLAST searches. Similar relative abundances of Bacteria and Archaea were observed in the microbiome of the sponge *C. concentrica* (85 and 1.6%, respectively), and substantial amounts of microbial coding sequences (92 Mb) and 16 rRNA gene clones (more than 3,000 clones) were produced and analyzed in this work [13]. Finally, the low percentage of *A. brasiliensis*-derived reads aligning to the genome of *A. queenslandica* (∼0.3%) might reflect the large amount of non-coding DNA regions in these genomes and/or polyphyly in Haploscleridae.

The microbial diversity associated with *A. brasiliensis* included both aerobic ammonia-oxidizing (*Nitrosomonadales*, *Rodhocyclales*, and *Hydrogenophylales*) and anaerobic bacteria using methane and sulfur electron accepters (*Methylophylales* and *Chromatiales*). Such complexity reveals a diversity of niches and microbial metabolism pathways in this sponge. A significant portion of the microbiome appears to be species-specific, possibly arising from the establishment of microbes with metabolic features that are beneficial for the host [2], [3]. For example, the high frequency of *Burkholderiales* (∼24.5%) that we observed in the microbiome of *A. brasiliensis* raises the possibility that this type of microbe plays a role in improving the host sponge’s fitness. *Burkholderiales* are not commonly found in abundance in the microbiomes of sponges and other marine holobionts. An exception to this observation comes from a report of an OTU (OTUHg1) closely related to the *Burkholderiales* genus *Cupriavidus* that was found at high frequency in the metagenome of *Haliclona (gellius)*, another haplosclerid sponge [37]. The enrichment of *Burkholderiales* in the sponge was greatest for the genus *Burkholderia* (∼5.6% of the total taxonomical hits). *Burkholderia* encompasses highly versatile microorganisms, including species known as human/animal pathogens, rhizosphere colonizers, plant-growth promoters, and phytopathogens [38], [39]. Of particular note, the *B. cepacea* D12 strain was isolated from deep sea sediments in South China [40]. Therefore, it is possible that some *Burkholderia* strains, such as those identified here, may have co-evolved towards a symbiotic association with the sponge *A. brasiliensis*. This type of association could be favored because *A. brasiliensis* settles on the sea floor, representing a possible niche for *Burkholderia* species.

### The *A. brasiliensis* Microbiome is Enriched for Sequences Associated with Membrane Transport and One-carbon Metabolism

**Figure 6:**
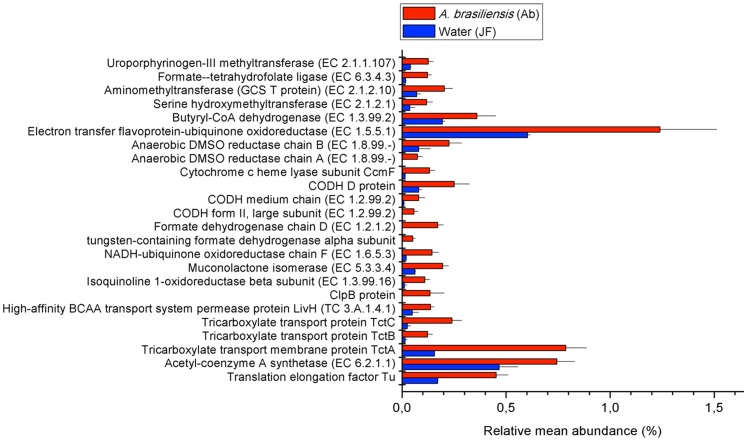
Functions enriched in the sponge that are predominantly associated with *Betaproteobacteria*, *Gammaproteobacteria*, and *Alphaproteobacteria*. The relative contributions of each function differed significantly (p-value <0.05) from the bacterial and/or total counts based on ANOVA for multiple groups.

The investigated sponge and seawater metagenomes differed significantly (p-value <0.00001) in terms of the relative abundances of almost half of the major subsystems they exhibited. The *Alphaproteobacteria* communities found in *A. brasiliensis* contributed relatively more than other sponge-associated bacteria to the overrepresentation of membrane transport-associated sequences, such as the tricarboxylate transport (TTT) system components TctA, TctB, and TctC (Figures 5 and 6). In the prototypical TTT system of *Salmonella typhimurium*, the periplasmic TTT-receptor TctC binds fluorocitrate, citrate, and isocitrate [41]. In the etiological agent of the whooping cough, *Bordetella pertussis*, the TctC ortholog BctC is present in the TTT system BtcABC and plays the role of a citrate importer [42]. Moreover, BctC was shown to be involved in the citrate-dependent up-regulation of the *bctABC* operon via interacting with the sensor protein BctE [43]. It is possible that sponge-associated *Alphaproteobacteria* respond and take up citrate and related tricarboxylate products available from the sponge by means of an orthologous TTT system.

With regard to respiration metabolism, sponge samples are enriched for carbon monoxide dehydrogenase (CODH), formate dehydrogenase (FDH), and anaerobic respiratory reductases, including anaerobic dimethyl-sulfoxide (DMSO) reductase (Figure 6). In a range of aerobic and anaerobic bacteria, CODH and FDH oxidoreductases catalyze the two-electron oxidation of carbon monoxide and the formate anion, respectively, producing carbon dioxide (CO_2_). In contrast, in the reductive acetyl-CoA pathway or the Wood–Ljungdahl (WL) pathway, nickel-containing CODH and tungsten-containing FDH catalyze the inverse reaction resulting in the reduction of two CO_2_ molecules, commonly using an anaerobic DMSO reductase (EC 1.8.99.-) as the terminal electron acceptor [44], [45], [46], [47]. CODH-bound carbonyl (*Western* branch) and cofactor-bound methyl (*Eastern* branch) residues derived from these CO_2_ reductions are then combined to form acetate through the acetyl-CoA synthase (ACS) activity of the CODH/ACS bi-functional complex [47]. Interestingly, the sponge samples were also enriched for uroporphyrinogen-III methyltransferase, a key enzyme involved in the synthesis of the WL pathway cofactor, vitamin B12 (Figure 6). It may be that the microbial communities associated with *A. brasiliensis* use the WL pathway for autotrophic carbon assimilation. In strictly anaerobic bacteria and Archaea, the WL pathway-associated Ni-CODH and W-FDH are extremely oxygen-sensitive enzymes. [48]. Another possibility is that *A. brasiliensis*-associated facultatively anaerobic *Proteobacteria* harbor oxygen-tolerant CODH and FDH enzymes to carry out thermodynamically favored oxidative processes involved in energy and CO_2_ generation.

### The *Arenosclera brasiliensis* Microbiome as a Source for the Synthesis and Degradation of Secondary Metabolites

Approximately 65% of the sequences related to secondary metabolic subsystems in the sponge (*n* = 110) were proteobacteria tryptophan (∼40%) and quinolinate (∼25%) synthases. Tryptophan and quinolinic acid are precursors of nicotinic acid in animals and bacteria, respectively. Nicotinic acid and/or nicotinic acid precursors produced by sponge-associated bacteria might be used in alkaloid (e.g., arenosclerins A-E) production in *A. brasiliensis*. Synthesis of quinolinic acid is expected to occur in a variety of microbes because it is the precursor of nicotinamide adenine dinucleotide (NAD), a crucial co-factor in bacteria [49]. However, there is no reason to predict that quinolinic acid synthesis would be relatively more abundant in the sponge-associated samples. Haplosclerid alkaloids are derived from piperidine-containing polyketide precursors, in which the piperidine moiety may be derived from a nicotinic acid moiety [50].

We hypothesize that the anti-microbial activity of arenosclerins may have induced the selection of tolerant microbes, shaping the species-specific microbial community diversity associated with *A. brasiliensis*. We found that sponge samples were enriched for proteobacterial isoquinoline 1-oxidoreductase (EC 1.3.99.16) and muconolactone isomerase (EC 5.3.3.4) (Figure 6). These enzymes are involved in the degradation of monocyclic aromatic compounds, including nitrogen-containing heterocyclic aromatic compounds, including piperidines. It is possible that certain *Proteobacteria* contained within the *A. brasiliensis* microbiome degrade arenosclerins, piperidine, or their precursors to achieve antibiotic resistance.

In this scenario, species belonging to the most abundant sponge-associated bacterial genus, *Burkholderia*, are strong candidates for regulating the production and/or degradation of polyketide-derived alkaloids extracted from *A. brasiliensis*. In the last five years, *Burkholderia* species have been shown to produce a diversity of bioactive polyketide-derived compounds, including i) rhizoxins, which are antimitotic macrolides produced by *B. rhizoxina*, the endosymbiote of the rice-pathogenic fungus *Rhizopus* sp. [51], [52]; ii) thailandamides and quorum-sensing regulated bactobolin antibiotics, produced by *B. thailandensis* [53]; iii) the food-poisoning toxin bongkrek acid, synthesized by *B. gladioli* [54]; and iv) the potent antibiotics the enacyloxins, produced by the *Burkholderia cepacea* complex (Bcc) species *B. ambifaria* [55]. In addition, *Burkholderia* are known to exhibit a special versatile mechanism for degrading natural or synthetic aromatic hydrocarbons, with some species being enriched for central and peripheral aromatic catabolic pathways [40], [56], [57].

### Concluding Remarks

This report provides the first large-scale analysis of the taxonomic and metabolic diversity of the microbiome of the sponge *A. brasiliensis*. Our results demonstrate that a complex microbiome exists within this sponge that presents a particular metabolic profile. We show that the *A. brasiliensis* microbiome is unique, differing from the microbiomes present in the water column surrounding these sponges and those associated with other marine organisms. Within the taxonomic signature of the *A. brasiliensis* microbiome, we detected an enrichment of *Betaproteobacteria* (e.g., *Burkholderia*) and *Gammaproteobacteria* (*Pseudomonas* and *Alteromonas*), indicating species specificity. Our results allowed us to speculate about diversity of niches in the sponge that might harbor anaerobes using methane and sulfur electron acceptors and those that might conduct thermodynamically favored oxidative processes involved in energy and CO_2_ generation. Our results may also suggest specific roles of *Burkholderia* sp. in this symbiosis. Finally, we hypothesized that secondary metabolites might have shaped the microbial community structure observed in *A. brasiliensis*. Studies are underway to uncover the diversity of polyketide synthase (PKS) genes and isolate possible sponge-specific (e.g., *Burkholderia*) symbionts from *A. brasiliensis*.

## Supporting Information

Figure S1 **Composition of the investigated metagenomes.** (A) Distribution of sponge and seawater taxonomic hits at the Domain hierarchical level, with sponge and seawater data represented by red and blue pie charts, respectively. (B) Sample rarefaction curves at the species hierarchical level.
(TIF)

Table S1 **Taxonomic profiles for the thirty-one analyzed marine-animal metagenomes.** Profiles were derived by BLASTn mapping of sequencing reads to the Genbank NT database and cumulatively summing the reads for higher-order clades (see Materials and Methods).
(XLSX)

Table S2 **Metagenomes overall numbers.** ^1^ - QC – MG-RAST 3.0 applied quality control of the reads. ^2^ - Values obtained considering the post QC metadata. Abbreviations: Seqs. – Sequences; Class. – Classifications.
(DOC)

Table S3 **Most abundant bacterial genera in sponge and water metagenomes.** ^1^– Relative percentage from the total number of organism classifications.
(DOC)

Table S4 **Functions overrepresented in the** ***A. brasiliensis*** **metagenomes.**
(DOC)

## References

[1] Love GD, Grosjean E, Stalvies C, Fike DA, Grotzinger JP (2009). Fossil steroids record the appearance of Demospongiae during the Cryogenian period.. Nature.

[2] Taylor MW, Radax R, Steger D, Wagner M (2007). Sponge-associated microorganisms: evolution, ecology, and biotechnological potential.. Microbiol Mol Biol Rev.

[3] Hentschel U, Hopke J, Horn M, Friedrich AB, Wagner M (2002). Molecular Evidence for a Uniform Microbial Community in Sponges from Different Oceans.. Applied and Environmental Microbiology.

[4] Webster NS, Taylor MW (2012). Marine sponges and their microbial symbionts: love and other relationships.. Environmental Microbiology.

[5] Vacelet J (1975). Etude en microscopie electronique de l’association entre bacteries et spongiaires du genre Verongia (Dictyoceratida).. J Microsc Biol Cell.

[6] Vacelet J, Donadey C (1977). Electron-microscope study of association between some sponges and bacteria.. J Exp Mar Biol Ecol.

[7] Fieseler L, Horn M, Wagner M, Hentschel U (2004). Discovery of the novel candidate phylum “Poribacteria” in marine sponges.. Applied and Environmental Microbiology.

[8] Sogin ML, Morrison HG, Huber JA, Mark Welch D, Huse SM (2006). Microbial diversity in the deep sea and the underexplored “rare biosphere”. Proceedings of the National Academy of Sciences of the United States of America.

[9] Lee OO, Wang Y, Yang J, Lafi FF, Al-Suwailem A (2010). Pyrosequencing reveals highly diverse and species-specific microbial communities in sponges from the Red Sea.. The ISME Journal.

[10] Webster NS, Taylor MW, Behnam F, Lucker S, Rattei T (2010). Deep sequencing reveals exceptional diversity and modes of transmission for bacterial sponge symbionts.. Environ Microbiol.

[11] Schmitt S, Tsai P, Bell J, Fromont J, Ilan M (2011). Assessing the complex sponge microbiota: core, variable and species-specific bacterial communities in marine sponges.. The ISME Journal.

[12] Schmitt S, Hentschel U, Taylor MW (2011). Deep sequencing reveals diversity and community structure of complex microbiota in five Mediterranean sponges.. Hydrobiologia.

[13] Thomas T, Rusch D, DeMaere MZ, Yung PY, Lewis M (2010). Functional genomic signatures of sponge bacteria reveal unique and shared features of symbiosis.. The ISME Journal.

[14] Turque AS, Cardoso AM, Silveira CB, Vieira RP, Freitas FAD (2008). Bacterial communities of the marine sponges Hymeniacidon heliophila and Polymastia janeirensis and their environment in Rio de Janeiro, Brazil.. Marine Biology.

[15] Turque AS, Batista D, Silveira CB, Cardoso AM, Vieira RP (2010). Environmental Shaping of Sponge Associated Archaeal Communities.. PLoS One.

[16] de Oliveira JHHL, Nascimento AM, Kossuga MH, Cavalcanti BC, Pessoa CO (2007). Cytotoxic Alkylpiperidine Alkaloids from the Brazilian Marine Sponge Pachychalina alcaloidifera.. J Nat Prod.

[17] Muricy G, Ribeiro SM (1999). Shallow-water Haplosclerida (Porifera, Demospongiae) from Rio de Janeiro State, Brazil (Southwestern Atlantic).. Beaufortia.

[18] Torres YR, Berlinck RG, Magalhaes A, Schefer AB, Ferreira AG (2000). Arenosclerins A-C and haliclonacyclamine E, new tetracyclic alkaloids from a Brazilian endemic Haplosclerid sponge Arenosclera brasiliensis.. J Nat Prod.

[19] Torres YR, Berlinck RG, Nascimento GG, Fortier SC, Pessoa C (2002). Antibacterial activity against resistant bacteria and cytotoxicity of four alkaloid toxins isolated from the marine sponge Arenosclera brasiliensis.. Toxicon.

[20] Stankevicins L, Aiub C, Maria LC, Lobo-Hajdu G, Felzenszwalb I (2008). Genotoxic and antigenotoxic evaluation of extracts from Arenosclera brasiliensis, a Brazilian marine sponge.. Toxicol In Vitro.

[21] Thompson FL, Bruce T, Gonzalez A, Cardoso A, Clementino M (2011). Coastal bacterioplankton community diversity along a latitudinal gradient in Latin America by means of V6 tag pyrosequencing.. Archives of Microbiology.

[22] Lôbo-Hajdu G, Guimarães ACR, Salgado A, Lamarão FRM, Vieiralves T (2004). Intragenomic, Intra- and Interspecific Variation inthe rDNA ITS of Porifera revealed by PCR-Single-Strand Conformation Polymorphism (PCR-SSCP).. Boll Mus Ist Biol Univ Genova.

[23] Margulies M, Egholm M, Altman WE, Attiya S, Bader JS (2005). Genome sequencing in microfabricated high-density picolitre reactors.. Nature.

[24] Meyer F, Paarmann D, D’Souza M, Olson R, Glass EM (2008). The metagenomics RAST server - a public resource for the automatic phylogenetic and functional analysis of metagenomes.. BMC bioinformatics.

[25] Parks DH, Beiko RG (2010). Identifying biologically relevant differences between metagenomic communities.. Bioinformatics.

[26] Altschul SF, Gish W, Miller W, Myers EW, Lipman DJ (1990). Basic local alignment search tool.. J Mol Biol.

[27] Srivastava M, Simakov O, Chapman J, Fahey B, Gauthier MEA (2010). The Amphimedon queenslandica genome and the evolution of animal complexity.. Nature.

[28] Bluman AG (2007). Elementary statistics: A step by step approach.. McGraw Hill Higher Education, New York, New York 6th edition.

[29] Benjamini Y, Hochberg Y (1995). Controlling the false discovery rate: a practical and powerful approach to multiple testing.. J Roy Stat Soc B.

[30] Kristiansson E, Hugenholtz P, Dalevi D (2009). ShotgunFunctionalizeR: an R-package for functional comparison of metagenomes.. Bioinformatics.

[31] Korbel JO, Snel B, Huynen MA, Bork P (2002). SHOT: a web server for the construction of genome phylogenies.. Trends Genet.

[32] Dutilh BE, Huynen MA, Bruno WJ, Snel B (2004). The Consistent Phylogenetic Signal in Genome Trees Revealed by Reducing the Impact of Noise.. Journal of Molecular Evolution.

[33] Dutilh BE, van Noort V, van der Heijden RTJM, Boekhout T, Snel B (2007). Assessment of phylogenomic and orthology approaches for phylogenetic inference.. Bioinformatics.

[34] Gascuel O (1997). BIONJ: An Improved Version of the NJ Algorithm Based on a Simple Model of Sequence Data.. Molecular Biology and Evolution.

[35] Felsenstein J (1989). PHYLIP - Phylogeny Inference Package (Version 3.2).. Cladistics.

[36] Wootters KW (1980). Statistical distance and Hilbert space.. Physical Review D.

[37] Sipkema D, Holmes B, Nichols SA, Blanch HW (2009). Biological Characterisation of Haliclona (?gellius) sp.: Sponge and Associated Microorganisms.. Microbial Ecology.

[38] Coenye T, Vandamme P (2003). Diversity and significance of Burkholderia species occupying diverse ecological niches.. Environmental Microbiology.

[39] Vial L, Chapalain A, Groleau M-C, Déziel E (2011). The various lifestyles of the Burkholderia cepacia complex species: a tribute to adaptation.. Environmental Microbiology.

[40] Wang Y, Yin B, Hong Y, Yan Y, Gu J-D (2008). Degradation of dimethyl carboxylic phthalate ester by Burkholderia cepacia DA2 isolated from marine sediment of South China Sea.. Ecotoxicology.

[41] Winnen B, Hvorup RN, Saier MH (2003). The tripartite tricarboxylate transporter (TTT) family.. Research in Microbiology.

[42] Antoine R, Jacob-Dubuisson F, Drobecq H, Willery E, Lesjean S (2003). Overrepresentation of a Gene Family Encoding Extracytoplasmic Solute Receptors in Bordetella.. Journal of Bacteriology.

[43] Antoine R, Huvent I, Chemlal K, Deray I, Raze D (2005). The Periplasmic Binding Protein of a Tripartite Tricarboxylate Transporter is Involved in Signal Transduction.. Journal of Molecular Biology.

[44] Drennan CL, Heo J, Sintchak MD, Schreiter E, Ludden PW (2001). Life on carbon monoxide: X-ray structure of Rhodospirillum rubrum Ni-Fe-S carbon monoxide dehydrogenase.. Proceedings of the National Academy of Sciences.

[45] Pierce E, Xie G, Barabote RD, Saunders E, Han CS (2008). The complete genome sequence of Moorella thermoacetica (f. Clostridium thermoaceticum).. Environ Microbiol.

[46] Raaijmakers H, Macieira S, Dias JM, Teixeira S, Bursakov S (2002). Gene sequence and the 1.8 A crystal structure of the tungsten-containing formate dehydrogenase from Desulfovibrio gigas.. Structure.

[47] Ragsdale SW, Pierce E (2008). Acetogenesis and the Wood-Ljungdahl pathway of CO(2) fixation.. Biochim Biophys Acta.

[48] Berg IA, Kockelkorn D, Ramos-Vera WH, Say RF, Zarzycki J (2010). Autotrophic carbon fixation in archaea.. Nat Rev Microbiol.

[49] Ollagnierdechoudens S, Loiseau L, Sanakis Y, Barras F, Fontecave M (2005). Quinolinate synthetase, an iron–sulfur enzyme in NAD biosynthesis.. Febs Letters.

[50] Fontana A (2006). Biogenetic Proposals and Biosynthetic Studies on Secondary Metabolites of Opisthobranch Molluscs.. Guido Cimino & Margherita Gavagnin (Eds): Molluscs From Chemo-ecological Study to Biotechnological Application Springer-Verlag Berlin Heidelberg.

[51] Partida-Martinez LP, Hertweck C (2005). Pathogenic fungus harbours endosymbiotic bacteria for toxin production.. Nature.

[52] Partida-Martinez LP, Hertweck C (2007). A gene cluster encoding rhizoxin biosynthesis in “Burkholderia rhizoxina”. Chembiochem.

[53] Nguyen T, Ishida K, Jenke-Kodama H, Dittmann E, Gurgui C (2008). Exploiting the mosaic structure of trans-acyltransferase polyketide synthases for natural product discovery and pathway dissection.. Nat Biotechnol.

[54] Rohm B, Scherlach K, Hertweck C (2010). Biosynthesis of the mitochondrial adenine nucleotide translocase (ATPase) inhibitor bongkrekic acid in Burkholderia gladioli.. Organic & Biomolecular Chemistry.

[55] Mahenthiralingam E, Song L, Sass A, White J, Wilmot C (2011). Enacyloxins Are Products of an Unusual Hybrid Modular Polyketide Synthase Encoded by a Cryptic Burkholderia ambifaria Genomic Island.. Chemistry & Biology.

[56] Chain PSG, Denef VJ, Konstantinidis KT, Vergez LM, Agullo L (2006). Inaugural Article: Burkholderia xenovorans LB400 harbors a multi-replicon, 9.73-Mbp genome shaped for versatility.. Proceedings of the National Academy of Sciences.

[57] O’Sullivan LA, Weightman AJ, Jones TH, Marchbank AM, Tiedje JM (2007). Identifying the genetic basis of ecologically and biotechnologically useful functions of the bacterium Burkholderia vietnamiensis.. Environmental Microbiology.

