# Supplementary figures and images for: Taxonomic and Functional Microbial Signatures of the Endemic Marine Sponge Arenosclera brasiliensis
Source: PLoS One. 2012 Jul 2;7(7):e39905. doi: 10.1371/journal.pone.0039905 (PMC3388064; doi:10.1371/journal.pone.0039905)

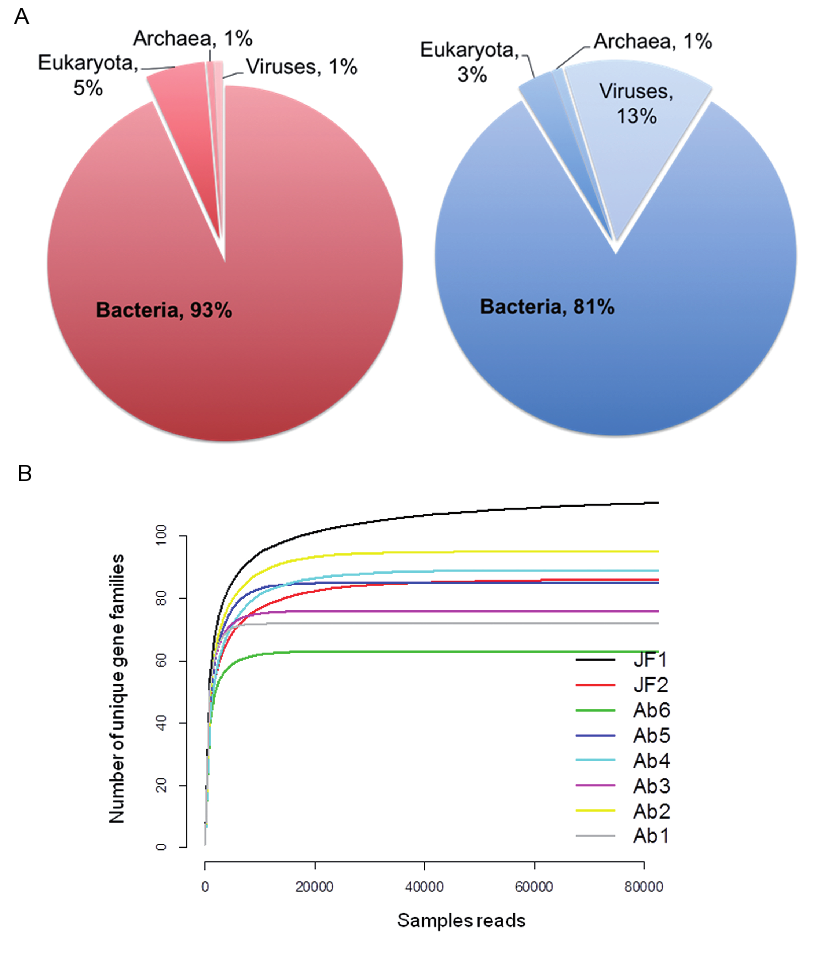

Supplement: Figure S1 — Composition of the investigated metagenomes. (A) Distribution of sponge and seawater taxonomic hits at the Domain hierarchical level, with sponge and seawater data represented by red and blue pie charts, respectively. (B) Sample rarefaction curves at the species hierarchical level. (TIF) [file pone.0039905.s001.tif]
