# Supplementary material for: Taxonomic and Functional Microbial Signatures of the Endemic Marine Sponge Arenosclera brasiliensis
Source: PLoS One. 2012 Jul 2;7(7):e39905. doi: 10.1371/journal.pone.0039905 (PMC3388064; doi:10.1371/journal.pone.0039905)
Supplement: Table S2 — Metagenomes overall numbers. 1 - QC – MG-RAST 3.0 applied quality control of the reads. 2 - Values obtained considering the post QC metadata. Abbreviations: Seqs. – Sequences; Class. – Classifications. (DOC) [file pone.0039905.s003.doc]

**Table S2 – Metagenomes overall numbers**

| Metagenome | Loaded Seqs. | Post QC^1^ | Size (bp)^2^ | Mean seqs.  Length (bp)^2^ | Mean GC % | Organisms  Class. (%)^2^ | Functional  Class. (%)^2^ | Hits vs *A. queenslandica* (%)^2^ |
| --- | --- | --- | --- | --- | --- | --- | --- | --- |
| Ab1 | 64 933 | 56 401 | 14 618 960 | 259 ± 97 | 43 ± 7 | 1 906 (3.4) | 3 087 (5.5) | 175 (0.31) |
| Ab2 | 167 475 | 142 408 | 39 867 206 | 279 ± 95 | 44 ± 7 | 8 171 (5.7) | 12 633 (8.9) | 430 (0.30) |
| Ab3 | 85 431 | 74 035 | 19 590 679 | 264 ± 96 | 43 ± 7 | 3 069 (4.7) | 5 102 (6.9) | 161 (0.22) |
| Ab4 | 133 157 | 113 070 | 32 072 840 | 283 ± 96 | 45 ± 8 | 9 697 (8.6) | 15 560 (13.8) | 341 (2.2) |
| Ab5 | 119 014 | 101 219 | 28 358 111 | 280 ± 94 | 43 ± 6 | 3 808 (3.8) | 6 530 (6.5) | 298 (0.29) |
| Ab6 | 71 969 | 62 161 | 16 363 344 | 263 ± 98 | 44 ± 7 | 3 776 (6.1) | 6 294 (10.1) | 200 (0.32) |
| JF1 | 112 267 | 85 742 | 37 322 982 | 435 ± 74 | 41 ± 10 | 41 633(48.4) | 50 747 (59.2) | 30 (0.03) |
| JF2 | 45 025 | 39 069 | 12 301 493 | 279 ± 96 | 41 ± 12 | 13 778 (35.3) | 20 631 (52.8) | 4 (0.01) |
| **Average** | **99 909** | **84 263 (~85%)** | **25 061 952** | **293 ± 93** | **44 ± 7 (Ab)**  **41 ± 11(JF)** | **5.54% (Ab)**  **44.40% (JF)** | **8.96% (Ab)**  **57.19% (JF)** | **0.3% (Ab)**  **0.02% (JF)** |

^1^ - QC – MG-RAST 3.0 applied quality control of the reads.

^2^ -Values obtained considering the post QC metadata.

Abbreviations: Seqs. – Sequences; Class. – Classifications.
