# Supplementary material for: Taxonomic and Functional Microbial Signatures of the Endemic Marine Sponge Arenosclera brasiliensis
Source: PLoS One. 2012 Jul 2;7(7):e39905. doi: 10.1371/journal.pone.0039905 (PMC3388064; doi:10.1371/journal.pone.0039905)
Supplement: Table S3 — Most abundant bacterial genera in sponge and water metagenomes. 1– Relative percentage from the total number of organism classifications. (DOC) [file pone.0039905.s004.doc]

**Table S3 – Most abundant bacterial genera in sponge and water metagenomes**

| *Arenosclera brasiliensis* (Ab) | | João Fernandinho’s seawater (JF) | |
| --- | --- | --- | --- |
| **Bacteria Genera** | **Mean abundace^1^**  **(# of hits)** | **Bacteria Genera** | **Mean abundace^1^**  **(# of hits)** |
| *Burkholderia* | 5.6 (1 709) | *Candidatus* Pelagibacter | 9.5 (5 258) |
| *Pseudomonas* | 3.6 (1 102) | *Synechococcus* | 5.3 (2 943) |
| *Synechococcus* | 2.4 (724) | *Prochlorococcus* | 2.2 (1 198) |
| *Roseobacter* | 2.0 (602) | *Pseudomonas* | 1.5 (828) |
| *Shewanella* | 1.9 (585) | *Roseobacter* | 1.2 (644) |
| *Vibrio* | 1.9 (580) | *Vibrio* | 1.1 (611) |
| *Ruegeria* | 1.7 (527) | *Bacteroides* | 1 (576) |
| *Neisseria* | 1.3 (405) | *Ruegeria* | 1 (538) |
| *Brucella* | 1.2 (373) | *Brucella* | 0.9 (511) |
| *Acidovorax* | 1.1 (344) | *Shewanella* | 0.9 (507) |

^1^ – Relative percentage from the total number of organism classifications.
