# Supplementary material for: Taxonomic and Functional Microbial Signatures of the Endemic Marine Sponge Arenosclera brasiliensis
Source: PLoS One. 2012 Jul 2;7(7):e39905. doi: 10.1371/journal.pone.0039905 (PMC3388064; doi:10.1371/journal.pone.0039905)
Supplement: Table S4 — Functions overrepresented in the A. brasiliensis metagenomes. (DOC) [file pone.0039905.s005.doc]

**Table S4 – Functions overrepresented in the *A. brasiliensis* metagenomes**

| **Function** | **Coefficient** | **AIC** | **Adjusted P-value** |
| --- | --- | --- | --- |
| Cysteine desulfurase (EC 2.8.1.7) | -0,7729849 | 208,97491 | 6,38E-71 |
| Cysteine desulfurase (EC 2.8.1.7), IscS subfamily | -1,7372151 | 154,53824 | 6,80E-64 |
| Phosphoenolpyruvate synthase (EC 2.7.9.2) | -2,5814688 | 80,18172 | 6,04E-63 |
| Tricarboxylate transport membrane protein TctA | -1,6208686 | 82,80667 | 1,53E-60 |
| Aconitate hydratase (EC 4.2.1.3) | -0,9737715 | 93,65574 | 8,39E-44 |
| PF00070 family, FAD-dependent NAD(P)-disulphide oxidoreductase | -22,4885634 | 41,12249 | 2,28E-38 |
| Methylcrotonyl-CoA carboxylase carboxyl transferase subunit (EC 6.4.1.4) | -1,8980304 | 73,66244 | 1,36E-34 |
| Carbon-monoxide dehydrogenase form II, large subunit (EC 1.2.99.2) | -22,31421 | 42,18567 | 2,91E-32 |
| TRAP-type C4-dicarboxylate transport system, large permease component | -1,8617041 | 53,41305 | 6,70E-32 |
| Phosphate acetyltransferase (EC 2.3.1.8) | -1,5069541 | 104,18425 | 6,70E-32 |
| Methylmalonate-semialdehyde dehydrogenase (EC 1.2.1.27) | -1,7265198 | 54,71138 | 1,72E-31 |
| 3-hydroxyisobutyrate dehydrogenase (EC 1.1.1.31) | -2,1426802 | 214,34677 | 2,17E-30 |
| Sulfite reductase [NADPH] hemoprotein beta-component (EC 1.8.1.2) | -21,240102 | 49,10492 | 3,87E-30 |
| L-serine dehydratase (EC 4.3.1.17) | -3,1853848 | 77,40865 | 4,48E-30 |
| Butyryl-CoA dehydrogenase (EC 1.3.99.2) | -0,7216479 | 201,22975 | 6,55E-29 |
| ATP-dependent DNA helicase RecQ | -2,2282721 | 116,26806 | 8,83E-29 |
| Tricarboxylate transport protein TctC | -2,252287 | 72,62042 | 7,32E-27 |
| Isoquinoline 1-oxidoreductase beta subunit (EC 1.3.99.16) | -20,0730479 | 118,68877 | 1,27E-25 |
| Sarcosine dehydrogenase (EC 1.5.99.1) | -1,5171064 | 81,29429 | 1,60E-25 |
| ClpB protein | -1,166904 | 85,95351 | 4,62E-25 |
| Maltose/maltodextrin ABC transporter, substrate binding periplasmic protein MalE | -22,0422763 | 42,87189 | 6,95E-25 |
| DNA topoisomerase III, Burkholderia type (EC 5.99.1.2) | -22,0422763 | 37,14083 | 6,95E-25 |
| Sarcosine oxidase alpha subunit (EC 1.5.3.1) | -0,7731008 | 149,64873 | 1,44E-24 |
| Hydroxymethylglutaryl-CoA lyase (EC 4.1.3.4) | -22,0265279 | 37,93389 | 1,59E-24 |
| N-acetylmuramoyl-L-alanine amidase (EC 3.5.1.28) | -21,0265279 | 52,43665 | 1,59E-24 |
| Betaine aldehyde dehydrogenase (EC 1.2.1.8) | -3,3423886 | 56,94525 | 2,42E-24 |
| Omega-amino acid--pyruvate aminotransferase (EC 2.6.1.18) | -1,4192931 | 97,82824 | 8,25E-23 |
| S-(hydroxymethyl)glutathione dehydrogenase (EC 1.1.1.284) | -2,297265 | 48,29787 | 2,09E-22 |
| Serine hydroxymethyltransferase (EC 2.1.2.1) | -0,6208702 | 278,54176 | 6,44E-22 |
| Aldehyde dehydrogenase (EC 1.2.1.3) | -0,8385937 | 108,94358 | 1,14E-21 |
| Predicted L-lactate dehydrogenase, Iron-sulfur cluster-binding subunit YkgF | -3,83771 | 40,02563 | 1,47E-21 |
| Acetoacetyl-CoA synthetase (EC 6.2.1.16) | -1,2023224 | 173,92834 | 1,06E-20 |
| Methylglutaconyl-CoA hydratase (EC 4.2.1.18) | -19,8346369 | 80,67036 | 2,49E-20 |
| Beta N-acetyl-glucosaminidase (EC 3.2.1.52) | -20,7954162 | 51,85678 | 1,50E-19 |
| Chaperone protein HscA | -2,2690941 | 48,31098 | 1,63E-18 |
| Uroporphyrinogen-III methyltransferase (EC 2.1.1.107) | -1,981412 | 48,62307 | 4,09E-18 |
| Isovaleryl-CoA dehydrogenase (EC 1.3.99.10) | -1,7890402 | 53,39478 | 9,36E-18 |
| COG0398: uncharacterized membrane protein | -4,2637944 | 36,93233 | 2,56E-17 |
| Translation elongation factor Tu | -0,9706305 | 61,92701 | 3,36E-17 |
| TldD protein, part of proposed TldE/TldD proteolytic complex (PMID 12029038) | -1,4464889 | 78,6157 | 6,01E-17 |
| Phosphate transport system permease protein PstC (TC 3.A.1.7.1) | -1,6712571 | 80,1885 | 2,06E-16 |
| Xanthine dehydrogenase, molybdenum binding subunit (EC 1.17.1.4) | -1,3274856 | 66,18078 | 5,74E-16 |
| Iron binding protein IscA for iron-sulfur cluster assembly | -20,5722726 | 51,20918 | 9,38E-16 |
| FIG001341: Probable Fe(2+)-trafficking protein YggX | -20,5722726 | 54,83602 | 9,38E-16 |
| Thiamin biosynthesis protein ThiC | -1,6041178 | 47,30385 | 3,63E-15 |
| Aminomethyltransferase (glycine cleavage system T protein) (EC 2.1.2.10) | -1,1743206 | 139,44274 | 5,17E-15 |
| Anaerobic dimethyl sulfoxide reductase chain A (EC 1.8.99.-) | -2,2437763 | 52,30786 | 5,46E-15 |
| Sarcosine oxidase beta subunit (EC 1.5.3.1) | -0,8488982 | 81,60629 | 1,19E-14 |
| tungsten-containing formate dehydrogenase alpha subunit | -1,9955967 | 44,45671 | 1,56E-14 |
| Peptidyl-prolyl cis-trans isomerase ppiB (EC 5.2.1.8) | -2,8977028 | 46,35248 | 2,08E-14 |
| Anaerobic dimethyl sulfoxide reductase chain B (EC 1.8.99.-) | -21,4669121 | 39,91857 | 3,02E-14 |
| Low molecular weight protein tyrosine phosphatase (EC 3.1.3.48) | -19,4669121 | 90,91397 | 3,02E-14 |
| Uracil-xanthine permease | -3,4400271 | 33,12308 | 6,71E-14 |
| DNA mismatch repair protein MutL | -20,4387412 | 43,47376 | 6,90E-14 |
| Xanthine dehydrogenase iron-sulfur subunit (EC 1.17.1.4) | -1,9524245 | 62,05982 | 1,18E-13 |
| Formate--tetrahydrofolate ligase (EC 6.3.4.3) | -1,0651213 | 64,10061 | 1,51E-13 |
| probable iron binding protein from the HesB_IscA_SufA family | -21,4097537 | 30,99155 | 1,62E-13 |
| Putrescine transport ATP-binding protein PotG (TC 3.A.1.11.2) | -20,4097537 | 41,90798 | 1,62E-13 |
| Xanthine dehydrogenase, FAD binding subunit (EC 1.17.1.4) | -2,5314584 | 62,3204 | 1,62E-13 |
| Tricarboxylate transport protein TctB | -2,1637336 | 48,05883 | 1,87E-13 |
| NADP-specific glutamate dehydrogenase (EC 1.4.1.4) | -0,9741495 | 115,947 | 2,81E-13 |
| N-carbamoylputrescine amidase (3.5.1.53) | -2,8143212 | 71,0581 | 4,57E-13 |
| Dihydrolipoamide acetyltransferase component of pyruvate dehydrogenase complex (EC 2.3.1.12) | -1,137442 | 127,28751 | 6,91E-13 |
| Muconolactone isomerase (EC 5.3.3.4) | -2,2815166 | 56,32745 | 8,28E-13 |
| Guanine deaminase (EC 3.5.4.3) | -2,2815166 | 54,88067 | 8,28E-13 |
| Alkyl hydroperoxide reductase subunit C-like protein | -1,5883695 | 69,16155 | 8,29E-13 |
| Fructose-bisphosphate aldolase class II (EC 4.1.2.13) | -1,5759469 | 65,17691 | 1,56E-12 |
| Cytochrome c heme lyase subunit CcmF | -1,1411072 | 87,98247 | 2,21E-12 |
| Pyruvate dehydrogenase E1 component (EC 1.2.4.1) | -0,646411 | 104,02565 | 3,17E-12 |
| 2-C-methyl-D-erythritol 2,4-cyclodiphosphate synthase (EC 4.6.1.12) | -20,2845906 | 50,02551 | 5,17E-12 |
| COG0009 Sua5 subfamily, required for N6-threonylcarbamoyl adenosine t(6)A37 modification in tRNA | -20,2845906 | 65,11814 | 5,17E-12 |
| L-fuconate dehydratase (EC 4.2.1.68) | -2,7233494 | 78,23716 | 1,05E-11 |
| Uncharacterized ABC transporter, permease component YrbE | -22,250689 | 29,90641 | 1,22E-11 |
| Putative stomatin/prohibitin-family membrane protease subunit YbbK | -1,7582685 | 80,69794 | 1,60E-11 |
| Carbon monoxide dehydrogenase medium chain (EC 1.2.99.2) | -21,2155977 | 36,53735 | 2,85E-11 |
| Glutamate--cysteine ligase (EC 6.3.2.2), divergent, of Alpha- and Beta-proteobacteria type | -20,2155977 | 51,86104 | 2,85E-11 |
| Beta-galactosidase (EC 3.2.1.23) | -19,2155977 | 94,91329 | 2,85E-11 |
| DinG family ATP-dependent helicase YoaA | -2,1637336 | 57,39842 | 6,61E-11 |
| Various polyols ABC transporter, ATP-binding component | -2,3644043 | 42,61161 | 6,66E-11 |
| Ferrochelatase, protoheme ferro-lyase (EC 4.99.1.1) | -1,1929547 | 59,81926 | 7,83E-11 |
| 3-ketoacyl-CoA thiolase [isoleucine degradation] (EC 2.3.1.16) | -2,8568808 | 37,30098 | 1,73E-10 |
| histone acetyltransferase, ELP3 family | -2,3178843 | 64,38661 | 3,01E-10 |
| Betaine--homocysteine S-methyltransferase (EC 2.1.1.5) | -1,5004394 | 141,29484 | 3,94E-10 |
| Formate dehydrogenase chain D (EC 1.2.1.2) | -21,102269 | 29,45799 | 4,01E-10 |
| Radical SAM family enzyme, similar to coproporphyrinogen III oxidase, oxygen-independent, clustered with nucleoside-triphosphatase RdgB | -1,5506291 | 148,12636 | 4,13E-10 |
| NADH-ubiquinone oxidoreductase chain F (EC 1.6.5.3) | -1,1521327 | 47,43072 | 5,97E-10 |
| Electron transfer flavoprotein-ubiquinone oxidoreductase (EC 1.5.5.1) | -1,0384531 | 97,16899 | 7,22E-10 |
| Magnesium and cobalt efflux protein CorC | -21,061447 | 32,7831 | 9,21E-10 |
| Hydroxymethylglutaryl-CoA synthase (EC 2.3.3.10) | -20,061447 | 65,05481 | 9,21E-10 |
| Gluconate dehydratase (EC 4.2.1.39) | -20,061447 | 39,47548 | 9,21E-10 |
| RNA polymerase sigma factor RpoH | -1,5759469 | 70,57418 | 1,41E-09 |
| carbon monoxide dehydrogenase D protein | -2,2437763 | 61,44582 | 2,67E-09 |
| NAD-dependent protein deacetylase of SIR2 family | -1,0186013 | 151,13338 | 4,10E-09 |
| TRAP-type C4-dicarboxylate transport system, periplasmic component | -1,2023224 | 44,62169 | 1,12E-08 |
| Acetyl-coenzyme A synthetase (EC 6.2.1.1) | -0,466465 | 85,14634 | 1,18E-08 |
| Pre-mRNA splicing factor PRP8 | -19,9279156 | 66,1797 | 1,29E-08 |
| 3-hydroxybutyryl-CoA dehydrogenase (EC 1.1.1.157) | -1,1257459 | 160,80906 | 1,31E-08 |
| Various polyols ABC transporter, permease component 1 | -3,59085 | 36,8973 | 1,46E-08 |
| Glutamate 5-kinase (EC 2.7.2.11) | -0,8419778 | 127,90453 | 2,65E-08 |
| Various polyols ABC transporter, permease component 2 | -21,8791255 | 23,99551 | 3,05E-08 |
| Fructose-1,6-bisphosphatase, type I (EC 3.1.3.11) | -19,8791255 | 50,53142 | 3,05E-08 |
| Prolipoprotein diacylglyceryl transferase (EC 2.4.99.-) | -19,8791255 | 59,48857 | 3,05E-08 |
| Methylmalonate-semialdehyde dehydrogenase (EC 1.2.1.27) | -1,5506291 | 61,87684 | 3,11E-08 |
| Nitrate/nitrite transporter | -1,5506291 | 45,69261 | 3,11E-08 |
| Predicted nucleoside ABC transporter, ATP-binding component | -0,7774392 | 55,23883 | 3,13E-08 |
| Glucose-1-phosphate cytidylyltransferase (EC 2.7.7.33) | -3,550028 | 36,46387 | 3,28E-08 |
| Na+/H+ antiporter NhaA type | -1,9560942 | 48,05453 | 3,71E-08 |
| Twitching motility protein PilT | -2,6406577 | 32,69893 | 4,09E-08 |
| D-2-hydroxyglutarate dehydrogenase | -2,419667 | 37,24423 | 4,80E-08 |
| 2-methylcitrate synthase (EC 2.3.3.5) | -1,219272 | 66,94219 | 5,07E-08 |
| 5&#39;-methylthioadenosine phosphorylase (EC 2.4.2.28) | -1,4705864 | 61,88156 | 5,23E-08 |
| Holliday junction DNA helicase RuvB | -1,3210547 | 60,03 | 5,79E-08 |
| L-aspartate oxidase (EC 1.4.3.16) | -1,0651213 | 64,47116 | 6,56E-08 |
| Citrate synthase (si) (EC 2.3.3.1) | -0,8274497 | 84,70255 | 8,04E-08 |
| Quinolinate synthetase (EC 2.5.1.72) | -0,8952223 | 95,82341 | 8,64E-08 |
| Ornithine aminotransferase (EC 2.6.1.13) | -1,8383112 | 59,3485 | 1,17E-07 |
| TRAP-type uncharacterized transport system, fused permease component | -1,6136873 | 47,16092 | 1,45E-07 |
| Rare lipoprotein A precursor | -21,7737649 | 22,89783 | 1,69E-07 |
| RNA polymerase sigma-54 factor RpoN | -19,7737649 | 57,68682 | 1,69E-07 |
| Proline dehydrogenase (EC 1.5.99.8) (Proline oxidase) | -0,9026024 | 72,57444 | 1,73E-07 |
| 3-isopropylmalate dehydratase large subunit (EC 4.2.1.33) | -0,8892306 | 66,2513 | 1,85E-07 |
| Various polyols ABC transporter, periplasmic substrate-binding protein | -2,0767222 | 36,45399 | 1,95E-07 |
| ComM-related protein | -2,3529756 | 37,24171 | 2,09E-07 |
| MG(2+) CHELATASE FAMILY PROTEIN | -2,3529756 | 37,24171 | 2,09E-07 |
| n-type ATP pyrophosphatase superfamily / TilS and TtcA-like | -2,8568808 | 64,18578 | 2,99E-07 |
| tmRNA-binding protein SmpB | -1,2474429 | 133,3902 | 3,35E-07 |
| ABC-type sugar transport system, periplasmic binding protein YcjN | -3,4164966 | 27,86492 | 3,76E-07 |
| Glutamine synthetase type I (EC 6.3.1.2) | -0,4897572 | 66,69647 | 3,93E-07 |
| Glutamyl-tRNA(Gln) amidotransferase subunit B (EC 6.3.5.7) | -0,8698126 | 81,17607 | 4,11E-07 |
| L-proline glycine betaine binding ABC transporter protein ProX (TC 3.A.1.12.1) | -1,8478807 | 49,48011 | 5,44E-07 |
| Altronate hydrolase (EC 4.2.1.7) | -1,1635614 | 67,24512 | 5,69E-07 |
| ATP-dependent protease La (EC 3.4.21.53) Type I | -0,6152044 | 59,48643 | 6,20E-07 |
| Muconolactone isomerase (EC 5.3.3.4),putative | -2,2815166 | 39,33459 | 9,04E-07 |
| Dihydroxy-acid dehydratase (EC 4.2.1.9) | -0,9698111 | 53,65695 | 1,15E-06 |
| Phosphate transport system permease protein PstA (TC 3.A.1.7.1) | -1,3528034 | 71,10777 | 1,62E-06 |
| Threonine dehydratase biosynthetic (EC 4.3.1.19) | -1,5205968 | 37,3781 | 1,66E-06 |
| Aspartyl-tRNA(Asn) amidotransferase subunit B (EC 6.3.5.6) | -0,8298072 | 80,68831 | 2,05E-06 |
| Ribokinase (EC 2.7.1.15) | -19,5914434 | 50,83355 | 2,23E-06 |
| tRNA pseudouridine synthase B (EC 4.2.1.70) | -19,5914434 | 49,34813 | 2,23E-06 |
| DNA mismatch repair protein MutS | -1,1341142 | 39,35306 | 2,28E-06 |
| Lysyl-tRNA synthetase (class II) (EC 6.1.1.6) | -1,17845 | 43,44614 | 2,72E-06 |
| Phosphate transport ATP-binding protein PstB (TC 3.A.1.7.1) | -1,0225617 | 86,464 | 2,79E-06 |
| High-affinity branched-chain amino acid transport system permease protein LivH (TC 3.A.1.4.1) | -1,0213187 | 54,57958 | 4,19E-06 |
| Enoyl-CoA hydratase [branched-chain amino acid degradation] (EC 4.2.1.17) | -3,2623459 | 27,49379 | 4,48E-06 |
| Transcription-repair coupling factor | -0,6015486 | 136,7344 | 5,06E-06 |
